# Berberine in Non-Alcoholic Fatty Liver Disease—A Review

**DOI:** 10.3390/nu14173459

**Published:** 2022-08-23

**Authors:** Anna Koperska, Agnieszka Wesołek, Małgorzata Moszak, Monika Szulińska

**Affiliations:** 1 Department of Treatment of Obesity, Metabolic Disorders and Clinical Dietetics, Poznan University of Medical Sciences, Szamarzewskiego 82/84, 60-569 Poznan, Poland; 2 Doctoral School, Poznan University od Medical Sciences, Fredry St. 10, 61-701 Poznan, Poland

**Keywords:** NAFLD, berberine, obesity, metabolic disorders

## Abstract

The incidence of Non-Alcoholic Fatty Liver Disease (NAFLD) has been rapidly increasing during the last decade. It is a relevant health problem that affects 25% of the general population. NAFLD involves an extensive array of clinical conditions. So far, no approved pharmacological therapy for NAFLD has been developed. Multiple bioactive compounds have been proposed to treat NAFLD. One of the most promising is Berberine (BBR). Its pleiotropic effect positively impacts various cardiometabolic aspects. In this review, we summarize NAFLD, its metabolic and cardiovascular complications, the hepatoprotective effects of BBR due to its broad spectrum of pharmacological effects, and the potential role of BBR in NAFLD therapy. BBR ameliorates NAFLD by affecting numerous abnormalities. It inhibits lipogenesis and gluconeogenesis, improves insulin resistance and lipid profile, and modulates gut microbiota. The exact mechanism underlying these effects is not yet entirely explained. A growing amount of evidence confirming the positive effects of BBR on multiple metabolic pathways, such as lipids and glucose metabolism, energy homeostasis, or gut microbiota modulation, allows us to speculate about the importance of this natural bioactive substance for NAFLD therapy.

## 1. Non-Alcoholic Fatty Liver Disease

### 1.1. NAFLD as a Clinical and Epidemiological Problem

NAFLD is nowadays a leading cause of chronic liver diseases and a significant public health concern worldwide. The disease affects 25% of the general population. Moreover, it is strongly related to progressive epidemics of obesity. Current data shows that 90% of NAFLD patients are obese, and about 70% of them have type 2 diabetes (T2D) or insulin resistance (IR) [1,2]. NAFLD is characterized by hepatocyte steatosis as a consequence of excessive lipid accumulation induced by metabolic factors, excluding alcohol intake and other factors damaging the liver. Suppose the liver is steatotic due to IR’s leading causes, such as obesity and a sedentary lifestyle [3]. In that case, it produces an excessive amount of glucose and triglycerides (TG), leading to low high-density lipoprotein (HDL) cholesterol levels and hyperinsulinemia [4]. This disease involves a wide scope of hepatic injuries ranging from simple steatosis (non-alcoholic fatty liver, NAFL) to non-alcoholic steatohepatitis (NASH) and cirrhosis [4,5]. Many factors contribute to the progression of NAFLD [6]. NAFL increases the risk of liver fibrosis [7]. The latter features predispose to T2D and cardiovascular diseases (CVD) [4]. Some patients with NAFLD may develop NASH, which is defined by persistent hepatocyte inflammation and damage with or without fibrosis. NASH may further progress to cirrhosis and liver cancer [8]. Clinical data indicate that approximately 1/3 of NAFL patients progress into NASH. When their condition develops into NASH, the risks of liver cirrhosis as well as hepatocellular carcinoma (HCC), and liver failure may rise significantly [9]. HCC is the most frequent type of primary liver cancer and an essential cause of solid tumor death [5]. As a result of the growing incidence and growth rate of NAFLD, NASH-derived HCC is also rising. In nearest years, NASH-related hepatic failure has been estimated to be the leading reason for liver transplantation [6]. NASH-HCC is more progressive and dangerous than viral hepatitis-related HCC, with a worse prognosis [5].

NAFLD is defined as follows: proven hepatic steatosis (HS), either obtained by imaging or histology, and a lack of secondary causes of HS, e.g., alcohol abuse, prolonged intake of steatogenic medications, or monogenic hereditary disorders, autoimmune hepatitis, hemochromatosis, Wilson’s disease [10,11].

The gold standard for NAFLD and NASH diagnosis remains the liver biopsy. The NAFLD’s diagnostic, however, is principally made by ultrasounds after other causes of chronic liver disease and alcohol intake of less than 20 g/day have been excluded. Applying validated scores, such as the fatty liver index (FLI) or fibrosis score, is also noteworthy [12].

The mild forms of NAFLD are asymptomatic, while NASH is refractory to therapy [13]. Currently, it is widely known that NAFLD is not a single-target disease. Pathogenesis of NAFLD involves impairment of lipid metabolism, endoplasmic reticulum (ER) stress, activation of inflammation, IR, leptin resistance, oxidative stress, and dysbiosis of the gut microbiota (GM) [6,9,14].

Metabolic abnormalities affect the progression of steatosis and contribute to Metabolic Syndrome (MetS) and NAFLD pathogenesis. Metabolic-related fatty liver disease (MAFLD) is a recent term, used for the first time in 2020, and was proposed to replace NAFLD. Contrary to NAFLD, MAFLD diagnosis does not require excluding patients with excessive alcohol intake or other chronic liver diseases. The presence of metabolic abnormality is crucial for the diagnosis of MAFLD. It emphasizes the predominant function of metabolic abnormalities in the pathogenesis of fatty liver disease [15].

According to research data by Ye et al., around 40% of NAFLD patients are non-obese. In fact, obesity is a crucial determinant for NAFLD. Still, as epidemiological data shows, it cannot be a primary criterium for NAFLD screening because NAFLD affects patients across all body mass index (BMI) ranges. Non-obese individuals with NAFLD present metabolic disorders, e.g., diabetes, hypercholesterolemia, and hypertension, and are at high risk for premature mortality [16].

NAFLD is a complex disease with a multifactorial background. To better understand its clinical significance and the urgent need to find efficient therapy, it is important to point out insulin resistance and inflammation in the pathogenesis of NAFLD, as well as different comorbidities of NAFLD: CVD, lipid and glucose metabolism abnormalities, and the role of GM in hepatic steatosis. An explanation of these aspects will prove that a pleiotropic agent combined with lifestyle intervention is needed to target all NAFLD features.

The current review sums up current knowledge in the field. The data sources were Pubmed, Medline, Cochrane, and Web of Science. Reviews, original articles, and meta-analyses published in English were included. No limitations were placed on the study design. A search was performed using various combinations of the following words: “NAFLD”, “Nonalcoholic steatohepatitis”, “hepatic steatosis”, “NASH”, “Non-alcoholic Fatty Liver Disease”, “Berberine”, “BBR”, “obesity”, “metabolic syndrome”, and “cardiovascular disease”. The bibliography of selected manuscripts was searched to find additional relevant studies that were not obtained in the primary search. Titles and abstracts were screened to identify studies for full-text.

#### Insulin Resistance and Inflammation in Pathogenesis of Obese NAFLD

NAFLD is becoming the most frequent indication for a liver transplant; extensive research has been conducted to determine the potential molecular mechanisms responsible for disease development and to find a successful treatment [6].

Obesity positively correlated with the degree of NAFLD. It might be noticed by the high incidence of NASH and severe fibrosis up to cirrhosis in patients with morbid obesity, which is the most prevalent and well-studied cause of NAFLD. Liver fat accumulation plays a crucial role in IR etiology [17].

Visceral adiposity is a more suitable indicator of NAFLD than body weight, body fat, or BMI [18]. The newest data indicates a significant correlation between visceral adiposity and HS. Increased visceral adiposity tissue leads to elevated TG hydrolysis and free fatty acid (FFA) delivery to the liver, contributing to IR development [18]. IR, lipid accumulation within the hepatocytes, and lower insulin sensitivity are causes of decreased liver fatty acid oxidation and intra-hepatic accumulation of TG. The impact on IR results from the excessive production of adipose-derived cytokines and hormones, such as tumor necrosis factor-alpha (TNF-a), interleukin 6 (IL-6), C-reactive protein (CRP), leptin, and adiponectin, with the development of an inflammatory pathway that exaggerates lipotoxic hepatic injury. Adipocyte-derived inflammation progresses into an intrahepatic inflammation with hepatocyte damage, which is engaged in the progression of NAFLD to NASH and cirrhosis [17]. Elevated levels of FFA, intra-hepatic accumulation of TG, and the intensified production of diacylglycerol and other lipotoxins activate protein kinase C-delta (PKC-δ) and nuclear factor kinase-B (NF-kB), causing an inflammatory response in the liver and IR [17].

Low-grade inflammation accompanies metabolic diseases, among them NAFLD and visceral obesity, leading to a heightened release of pro-inflammatory cytokines, which promote the progression of IR and other complications associated with NAFLD [17]. The inflammation in the white adipose tissue was proposed as an activator of the NF-kB pathway in the liver. It is responsible for increasing the transcription of various pro-inflammatory genes, which may enlarge chronic systemic inflammation that leads to extrahepatic cellular injury [17].

### 1.2. Conditions Associated with NAFLD

#### 1.2.1. CVD Risk and Proatherogenic Lipid Profile

NAFLD patients are predisposed to cardiovascular (CV) morbidity and mortality. Modifying CVD risk factors should be obligatory in all patients with NAFLD [12]. Endothelial dysfunction contributes to the major cardiovascular problems, such as plaque initiation and progression, hypertension, chronic heart failure, and coronary artery disease, [19]. The degree of advancement of the liver disease appears to be related to the risk of CV events. Clinical and epidemiological data underlie NAFLD’s role in developing various CV manifestations, including left ventricular dysfunction, cardiac conduction system abnormalities, atherosclerotic CV disease, and ischemic stroke, indicating that its contribution may be separate from the occurrence of commonly known CV risk factors [20].

The exact mechanisms linking NAFLD with CVD have not been fully explained. However, it is generally agreed that disturbances in lipid accumulation in the liver, increased inflammation, and liver fibrosis are profound in conditions of CVD related to NAFLD [1,2]. Interrelation between the degree of HS on ultrasound and the CV risk, assessed by the Framingham Risk Score, has also been confirmed [21]. Considering the observed dependence between the ultrasonographic advancement of steatosis and coronary atherosclerosis, it might be assumed that liver ultrasound may aid in diagnosing individuals at high CV risk [20].

It should be stressed that NAFLD is related to a rise in components of the renin–angiotensin system, such as angiotensin II, which may be associated with vascular injury through higher oxidative stress and subsequent inhibition of the insulin signaling pathways, enhancing atherosclerosis. Moreover, the dysregulation of insulin signaling in the endothelium causes vasoconstriction that promotes arterial hypertension. Angiotensin II also escalates the NAFLD to NASH progression and may then result in fibrosis by stimulation of fibroblasts and induction of pro-inflammatory cytokines [17].

One of the standard features of NAFLD is the proatherogenic lipid profile, which comprises increased TG concentration, small-dense low-density lipoprotein (LDL), and decreased concentration of HDL. Atherogenic dyslipidemia is endothelial dysfunction and reno-vascular injury risk factor [17,22]. NAFLD, especially the NASH form, usually contributes to a boosted release of pro-coagulant, pro-oxidant, and pro-fibrogenic factors mediating other organ dysfunction [17]. High LDL concentration and oxidized LDL (oxLDL) are significant risk factors for endothelial dysfunction and atherosclerosis [23].

The evidence of an increase in CV risk in NAFLD indicates an urgent need to adopt early intervention to reduce the risk of disease progression and improve CV outcomes.

#### 1.2.2. Glucose Homeostasis and T2D in NAFLD

NAFLD is accompanied by impaired glucose metabolism. The liver plays a crucial role in glucose homeostasis. NAFLD is related to IR and the development of T2D [24].

It is well accepted that disturbances in lipid metabolism are critical to developing NAFLD and its progression. They can affect various reactive oxygen species (ROS) generators, including mitochondria, ER, and nicotinamide adenine dinucleotide phosphate (NADPH) oxidase (NOX). However, the contributing role of ROS generators to the development of NAFLD remains unverified. The latest scientific data show us how increased ROS levels affect fluctuations in insulin sensitivity, activities of relevant enzymes of glucose metabolism, immune system signaling pathways, and inflammatory response [14].

#### 1.2.3. Gut Microbiota and Its Role in Hepatic Steatosis

There is growing evidence that GM imbalance is an essential element of the pathogenesis and severity of HS. The intestinal microbiota and bacterial metabolites may play a role in the development of liver diseases through different mechanisms, usually connected with intestinal permeability triggering the release of lipopolysaccharide (LPS), cytokines, and gut microbiota DNA into the system circulation and to the liver, accelerating inflammation, production of short-chain fatty acids (SCFAs), and changes in metabolism [25].

#### 1.2.4. Comorbidities Linked to NAFLD

Other comorbidities associated with obesity, such as obstructive sleep apnea, hyperuricemia or hypo-testosteronemia in men, and polycystic ovary syndrome (PCOS) in women, seem to be involved in developing NAFLD, demonstrating a more complex relationship between obesity and hepatic functions [17]. Recently, the link between hypothyroidism and NAFLD has been proved. A meta-analysis of 13 prospective studies provides solid epidemiological evidence that shows how hypothyroidism may increase NAFLD risk to more than 50%. The risk increases to 70% if subclinical hypothyroidism is excluded [12]. Patients with hypothyroidism are at a higher risk of developing NAFLD than individuals with normal thyroid function [26]. Apart from being the most frequent cause of liver disease, data indicates that NAFLD is associated with a relevant increase in risk for T2D, metabolic syndrome, and CVD [27,28].

To summarize, NAFLD is a complex disorder with multiple comorbidities, so desirable therapy should efficiently fight against diversified components from liver fat accumulation and IR to vascular complications.

### 1.3. Lifestyle Interventions in the Treatment of NAFLD

The leading cause of primary NAFL and NAFLD are overweight and obesity. Excessive fat accumulation in the liver can lead to T2D and hypertriglyceridemia, which often accompany NAFLD. In the development of obesity, modifiable dietary-behavioral triggers (excess energy consumption, sedentary lifestyle) seem crucial. Considering those factors in prevention and the formulation of a treatment strategy for NAFLD should be a priority [12].

A sedentary lifestyle and overnutrition are modifiable factors that promote obesity and NAFLD. Lifestyle interventions dominate the treatment for NAFLD. A 5–7% weight reduction achieved by a net calorie restriction of 500–1000 kcal/day through diet calorie restriction and increased physical activity is recommended [3]. The problem is poor patient adherence to long-term modifications. Therefore, understanding the role of nutritional modulation of hepatic fat content and IR appears crucial to preventing and treating NAFLD. Hypocaloric diets decrease, whereas overfeeding and hypercaloric, high carbohydrate diets increase the liver fat content. For now, there is no sufficient evidence to prove fructose is more harmful than glucose, but fructose metabolism might have a more negative hepatic impact than glucose [4]. Consumption of sugared soft drinks heightens the risk of developing NAFLD [29]. A combination of dietary modifications and exercise is an essential treatment for NAFLD and NASH [30,31]. Physical activity contributes to the improvement of NAFLD via increased insulin sensitivity, decreased abdominal obesity, and hepatic fat [3,30]. It should be noted that the Mediterranean Diet is optimal for managing NAFLD and preventing CV complications because it is rich in antioxidants and fiber and is characterized by a favorable lipid profile and low content of simple sugars [32,33]. Also recommended is avoiding energy-dense foods, low in fiber, rich in red meat, trans fats, refined carbohydrates, and high-fructose corn syrup [7].

### 1.4. Pharmacological Therapy and Herbal Medicine

At present, no approved pharmacological therapy is available for NAFLD. New therapeutic targets should lead to the development of agents that reduce disease progression and related complications. Medicines are used to manage diabetes, hypercholesterolemia, or hypertension comorbidities [2]. Multiple clinical studies are testing candidates of NASH drugs, such as agonists of nuclear receptors (obeticholic acid, elafibranor), glucagon-like peptide-1 receptor agonists, and insulin sensitizers (glitazones, pioglitazone, metformin) [13]. Due to limited therapy regimes and efficacy, no drugs have been approved to treat NASH until now.

Promising effects of obeticholic acid (OCA) on NASH and its metabolic features have been reported. OCA is a selective farnesoid X receptor (FXR) agonist with anticholestatic and hepato-protective properties [34]. A study of patients with T2D and NAFLD showed that the administration of OCA was well tolerated and generated an increase in insulin sensitivity and a reduction of markers of liver inflammation and fibrosis [35].

Well-known traditional herbal medicines for treating NAFLD and NASH are, e.g., resveratrol, curcumin, and silymarin. It was concluded that an effective therapeutic agent should be able to modulate different pathways associated with the NAFL/NASH progression [6]. Thus, there is an urgent need to provide a therapy that would be safe and effective in NAFLD but also preventive towards progression or CV complication development.

## 2. Berberine

### 2.1. Berberine—General Information

BBR is an isoquinoline alkaloid initially isolated from the root and rhizome of the herb *Coptis chinensis*. It has been commonly used in traditional Chinese Medicine to treat diarrhea and many infectious gastrointestinal disorders for over 3000 years. BBR is present as a bioactive constituent in the roots, rhizomes, stem, and barks of many medicinally plants, e.g., *Hydrasti scanadensis* (goldenseal), *Berberis vulgaris* (barberry), *Berberis aquifolium* (Oregon grape), *Coptis chinensis* (Coptis or goldenthread), and *Berberis aristata* (Tree turmeric) [36,37]. However, BBR is currently produced by the process of chemical synthesis. The chloride or sulfate salt of BBR is widely used in clinical practice. It is an intense yellow powder with no smell and a characteristic alkaloidal bitter taste [36]. BBR has very low toxicity in usual doses and is clinically beneficial with the absence of significant side effects. The only reported adverse effects were mild gastrointestinal reactions that rarely occurred [38].

**Figure 1:**
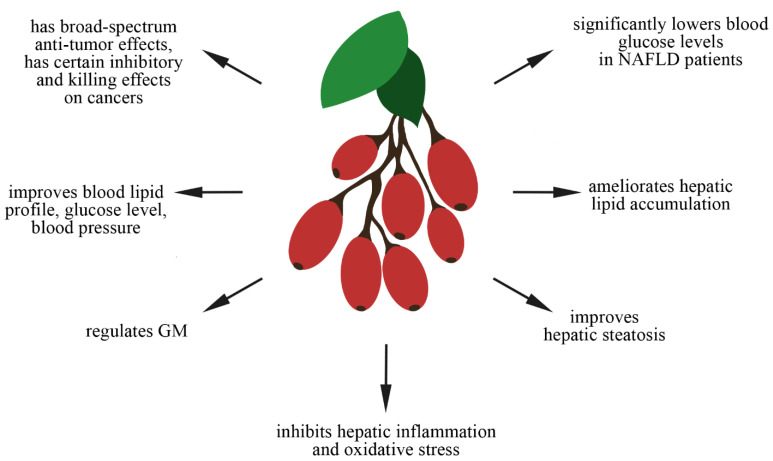
Actions of BBR.

Recently, multiple studies confirmed the positive role of BBR in preventing CV and metabolic disorders such as NAFLD and T2D [6]. Specific data are presented in Table 1. BBR is drawing much attention as a promising option for improving NAFLD and its complications. Over the years, clinical investigations on BBR have demonstrated a broad spectrum of pharmacological effects. Some data have highlight the crucial antihypertensive, antihyperglycemic, antioxidant, anti-inflammatory, and hypolipidemic activity of berberine. Various studies present the nephroprotective, hepatoprotective, and cardioprotective potential of BBR [32]. So far, BBR has been used as a non-prescription drug to treat diarrhea, stomatitis, dysentery, and hepatitis. Numerous studies have been conducted to investigate its other pharmacological properties and therapeutic efficiency, primarily on T2D, lipid metabolism, and tumors [33]. The summary of BBR properties is shown in Figure 1.

A meta-analysis of 27 randomized controlled trials (RCTs) on BBR reported no profound adverse effects during T2D, hyperlipidemia, or hypertension treatment. In addition, treatments with BBR in these trials were at a relatively low cost compared with other first-line medicines and therapies [38]. A meta-analysis of 21 clinical trials revealed that BBR is effective in the treatment of T2D, hyperlipidemia, and hypertension, in comparison with other therapeutic options [39]. Because MAFLD conception is gaining more attention, there is a need to provide studies assessing BBR use and efficacy in this specific group of patients. Considering metabolic disorders accompanying hepatic steatosis in MAFLD individuals, they may achieve positive outcomes due to including BBR in disease management. Currently, no clinical data refers to BBR in MAFLD, but it is a promising direction for future research. The effect of BBR on NAFLD might be more effective if more studies with longer terms were included. Available data are presented in Table 1.

**Table 1:** Effects of BBR presented in animal and clinical studies.

Reference	Study Type	Population	Intervention	Effects
Animal studies				
Xia et al., 2011 [40]	animal model study	The type 2 diabetic rat models, n = 9	380 mg·kg^−1^·d^−1^/1 day (BBR), 5 weeks	↓ glucose, FAS, body mass, TC, TG ↓ gluconeogenic genes, Phosphoenolpyruvate carboxykinase (PEPCK) and Glucose-6-phosphatase (G6Pase) in liver, ↓ hepatis steatosis
Ge et al., 2011 [41]	animal model study	primary hepatocytes from Sprague-Dawley (SD), Zucker lean (ZL) or fatty (ZF) rats	10, 25, 50 or 100 µM (Berberine hydrochloride in primary hepatocytes from Sprague-Dawley (SD), Zucker lean (ZL) or fatty (ZF) rats	BBR regulate genes involved in glucose and fatty acid synthesis in hepatocytes.
Zhang Z et al., 2014 [42]	animal model study	male mice and wild-type mice, n = 5	5 mg kg^−1^ per day (BBR). 4 weeks	↓ body weight, % fat mass of BW, serum FAA, blood glucose, glucose AUC, serum insulin
Li Zhao et al., 2017 [43]	animal model study	Sprague–Dawley rats, n = 6	150 mg/kg body weight/1 day. 16 weeks	↓ body mass, TG, LDL, HOMA-IR, and ↑ ISI, ↓ Ra_glu_, GNG and hepatic lipogenesis
Yixuan Sun et al., 2018 [44]	animal model study	Eight-week-old male C57BL mice, n = 6	5 mg·kg^−1^·day^−1^/1 day (BBR), 4 weeks	↓ liver TG, liver cholesterol, TG, plasma cholesterol, body weight
Yan Luo et al., 2019 [5]	animal model study	The C57BL/6J mice, n = 30	250 mg/kg/1 day, 12 weeks	↓ NAS, ALT, AST, glucose, HDL, LDL, TC
Human studies				
Kong et al., 2004 [45]	RCT	adult hypercholesterolemic patients, n = 63	1 g/1 day (BBR hydrochloride), 3 months	↓ ALT, AST, GGT, TC, TG, LDL-c
Xie et al., 2011 [46]	RCT	adult NAFLD and 2 diabetes patients; n = 60	0.3 g/1 day (BBR), 12 weeks	↓ TG, TC, LDL, ALT ↓ liver lipid content
Bai et al., 2011 [47]	RCT	adult NAFLD patients, n = 68	0.5 g/1 day (BBR + metformin) 3 months	↓ of level of FPG, TC, TG, LDL-C, FINS, HOMA-IR, ↑ adiponectin, ↓ IR
Marazzi et al., 2011 [48]	RCT	elderly hypercholesterolemic patients, n = 80	0.5 g/1 day (BBR + policosanol 10 mg, red yeast rice 200 mg, folic acid 0.2 mg, coenzyme Q10 2.0 mg, and astaxanthin 0.5 mg), 12 months	↓ TC, LDL-C, IR
Di Pierro et al., 2012 [49]	RCT	adult 2 diabetes patients; n = 22	0.588 g/1 day (Berberol^®^, *B. aristata* extract titered as 85% berberine and 105 mg of *S. marianum* extract titered as >60% flavonolignans), 90 days	↓ HbA1c, TC, LDL-C, HDL-C, T, FR, BMI, HOMA-IR
Cao et al., 2012 [50]	RCT	adult NAFLD patients, n = 78	0.5 g/1 day (BBR + metformin) 16 weeks	↓ HOMA-IR, TC, TG, LDL, ALT, AST, 2hPG
Pérez-Rubio et al., 2013 [51]	RCT	adult MetS patients, n = 24	1.5 g/1 day (berberine hydrochloride), 3 months	↓ SBP, waist circumference, TG, and total insulin secretion. ↓ waist circumference in females, SBP, TG, area under the curve (AUC) of glucose, AUC of insulin and insulinogenic index.
Ning et al., 2013 [52]	RCT	adult NAFLD patients; n = 44	0.5 g/1 day (BBR + metformin), 16 weeks	↓ HbA1C, TC, TG
Manzato & Benvenut, 2014 [53]	RCT	adult dyslipidemic patients, n = 1161	0.5 g/1 day BBR + ed yeast rice extract 200 mg (equivalent to 3 mg monacolins), policosanol 10 mg, 0.2 mg folic acid, coenzyme Q10 2 mg, and asthaxantin 0.5 mg (Armolipid Plus, Rottapharm|Madaus) with or without diet, 16 weeks	↓ TC, LDL-C, TG
Li, 2015 [54]	RCT	adult NAFLD patients, n = 96	0.3 g/1 day (BBR), 3 months	↓ 2hPG, HbA1C, TC, LDL, ALT, AST
Yan H-M. et al., 2015 [55]	RCT	adult NAFLD patients, n = 184	1.5 g/1 day (BBR), 16 weeks	↓ hepatic fat content, ALT, AST, y-GT, glucose, HOMA-IR, TC, TG, LDL-c
Wang et al., 2016 [56]	RCT	adult mild hyperlipemia patients, n = 97	0.3 g/1 day (vs. 0.9 g/1 day), 3 months	↓ TG, TC i LDL-C, ↑ HDL-C
Xinxia Chang et al., 2016 [24]	RCT	adult NAFLD patients, n = 80	1.5 g/1 day (BBR), 16 weeks	↓ TC, TG, LDL-c, glucose, HOMA-IR, ↓ hepatic fat content

Abbreviations: T2DM: Type 2 Diabetes; 2hPG: 2-h post-load plasma glucose; AUC: Area Under ROC Curve; BBR: Berberine; BMI: Body Mass Index; FFA: Free Fatty Acid; FAS: Fatty Acid Synthase; FINS: Fasting Insulin; FPG: Fasting Plasma Glucose concentration; G6Pase: Glucose-6-Phosphatase; GGT: Gamma-Glutamyl Transferase; GNG: Granulomatous Amebic Encephalitis; HbA1c: Glycated hemoglobin; HOMA-IR: Homeostatic Model Assessment—Insulin Resistance; IR: Insulin Resistance; ISI: Insulin Sensitivity Index; LDL: Low-Density Lipoprotein; NAS: NAFLD Activity Score; PEPCK: Phosphoenolpyruvate Carboxykinase; Ra_glu_: Glucose appearance; SD: Sprague-Dawley Rats; TC: Total Cholesterol; TG: Triglicerydes; y-GT: Gamma-glutamyltransferase; ZF: Zucker Fatty rats; ZL: Zucker Lean rats; ↓: lowered; ↑: elevated

### 2.2. Bioavailibillity and Pharmacokinetics

The bioavailability of BBR was proved to be less than 1%. However, its physicochemical properties, pharmacokinetics, and metabolism are not fully elucidated. The pharmacokinetics studies demonstrated that the liver contains the greatest concentration of BBR metabolites (50–70 times higher than the plasma levels) after oral administration [57]. The ameliorative NAFLD effect of BBR is likely linked to its favorable location in the liver and its direct impact on different hepatic genes involved in energy metabolism [58]. Moreover, the half-life of BBR in hepatic tissue lasts longer than in other tissues, which shows why the liver is the main target organ of BBR [9,36]. The newest studies have highlighted that CYP2D6 is the primary human cytochrome P450 (CYP) for the production of BBR metabolite, followed by CYP1A2, 3A4, 2E1, and CYP2C19 [38]. BBR affects drug metabolic enzyme activity and may play an essential role in drug interactions [59]. The poor oral bioavailability accounts for the low transport to blood, which is determined by several factors. Solubility is markedly decreased by drug self-aggregation. BBR aggregate, particularly at low pH, causes low absorption in the stomach and upper small intestine. The permeability of BBR is low. Eighty percent of BBR is metabolized in the liver and intestine by CYP2D6. It is relevant to enhance the bioavailability by improving the structure of the drug or intensifying permeation with additives [37]. BBR can penetrate the blood-brain barrier, and its metabolites are widely distributed in the liver, muscle, kidney, lung, heart, brain, pancreas, and adipose tissue. The organ distribution of BBR is fast, with maximum allocation in the liver and a minor distribution in fat [36]. The low bioavailability of BBR due to its low oral absorption and complex metabolism significantly limits its clinical use [59]. Scientific developments regarding polymer materials, nanotechnology, and particle engineering could eliminate some problems. Various types of nanocarriers encapsulating BBR can overcome these barriers [8]. Nanotechnology methods can improve BBR efficacy and bioavailability because of the characteristics of small particles, high surface reactivity, multiple active centers, and strong adsorption capacity. New solutions selectively target nano-drugs in the liver in order not to have an impact on the physiological functions of other organs. Studies of different BBR nanoparticles support new drug development [59].

### 2.3. The Role of BBR in Insulin Resistance

The induction of IR causes attenuation of the glucose-lowering effect of insulin. Insulin as an anabolic hormone increases lipogenesis, which leads to accelerated liver steatosis. BBR can improve the insulin signaling pathway. BBR affects IR by increasing insulin secretion and insulin receptor (InsR) expression via protein kinase C (PKC) activation, inducing glucose transporter (GLUT4) translocation to the cell membrane and lowering ER stress. The alleviation of IR by increasing message ribonucleic acid (mRNA) expression is one of the solutions in treating NAFLD with BBR [8]. BBR can improve patients’ insulin sensitivity through the PPAR-γ pathway and enhance tyrosine phosphorylation [9]. Adiponectin—adipokine secreted by adipocytes—increases insulin sensitivity through AMP-activated protein kinase (AMPK) [60]. BBR may improve IR by increasing the expression of adiponectin receptors. BBR increases insulin sensitivity due to its ability to upregulate InsR expression in a dose- and time-dependent manner, which promotes cellular glucose uptake in the presence of insulin [19]. BBR was reported to decrease fasting blood glucose (FBG), hemoglobin A1c (HbA1C), and insulin levels in T2D individuals at levels similar to metformin and rosiglitazone [19].

### 2.4. BBR Impact on Glucose Regulation

BBR, thanks to its properties, might be proposed as an anti-diabetes agent. BBR may exert a beneficial effect on NAFLD by remodeling glucose metabolism. BBR increases the amount of GLUT4, which can accelerate glucose ingestion and, consequently, reduce glucose concentration in the blood. Additionally, BBR inhibits intestinal glucose absorption, which is linked to the BBR glucose-lowering property. BBR substantially decreased FBG, HbA1C, TG, and insulin levels in individuals with T2D in the clinical study [24]. In the liver, IR manifests itself by excessive glucose production (gluconeogenesis) in the fasting state and impaired glucose uptake after a meal, despite the presence of insulin. IR exhibits lowered glucose uptake in muscles, leading to postprandial hyperglycemia [37]. Some evidence suggests that BBR combined with metformin effectively treats patients with NAFLD and T2D [50,52,60]. For instance, BBR could accelerate insulin secretion, ameliorate IR, inhibit gluconeogenesis, and promote glucose uptake [60]. Furthermore, through AMPK activation, BBR can stimulate glucose uptake in muscles, liver, and adipose and inhibit gluconeogenesis in the liver by downregulation of gluconeogenic enzymes [37]. The hypoglycemic effect of BBR encompasses the regulation of GM, activating the AMPK pathway, inducing intestinal glucagon-like protein-1 secretion, promoting glycolysis in peripheral tissue cells, and enhancing hepatic low-density lipoprotein receptor (LDLR) mRNA expression [38].

### 2.5. Berberine vs. Metformin

Metformin and BBR have many similar features, regardless of different structures. Both might be excellent drugs for the treatment of T2D, obesity, cardiac diseases, tumor, and inflammation [37]. Metformin lowers macrovascular complications in T2D patients [37]. It inhibits liver glucose production and promotes glucose uptake in muscle and adipose, improving hyperglycemia and hyperlipidemia and alleviating NAFLD. The primary target of metformin is the 5′-AMP-activated protein kinase (AMPK), but some effects seem to be mediated through mechanisms independent from AMPK [37]. In cases of intolerance or refractory to metformin, BBR appears to be a suitable alternative or additive agent to ensure tolerance and lower the risk of adverse effects [37]. Some studies underlie that metformin combined with BBR might be a beneficial therapeutic option [50,52,61]. Studies have reported that the synergistic action of BBR and metformin is due to similar anti-diabetic mechanisms, despite different transporters and metabolism. Combining these two drugs might reduce the dosage of the particular drug to overcome problems such as the oral bioavailability of BBR and adverse effects. BBR presents a high hypoglycemic potential; it has been proven to activate AMPK with subsequent glycolysis induction [23]. BBR is an effective insulin sensitizer with properties comparable to metformin [38].

### 2.6. Lipid Lowering Effects of BBR

Individuals with NAFLD present a proatherogenic lipid profile with elevated levels of TG and LDL, accompanied by low HDL concentration. The positive effect of BBR on NAFLD encompasses a direct regulation of lipid metabolism in the liver [58]. Recent studies have demonstrated the impact of BBR on lipid level. BBR exerted an antihyperlipidemic effect by lowering patients’ total cholesterol (TC), TG, and LDL levels. BBR has been reported to reduce oxLDL. The cholesterol-lowering mechanism of BBR was different from that of statins. BBR heightened the LDLR and blocking proprotein convertase subtilisin/kexin type 9 (PCSK9) transcription. PCSK9 modulates lipid homeostasis via degradation of LDLR and lowering LDL [9,23,24,37]. BBR metabolite also promotes the reduction of TG levels. It can aid the excretion of cholesterol from the liver to bile and, as a result, blood lipid can be decreased. Moreover, BBR lowers blood cholesterol levels by minimizing intestinal absorption, cholesterol uptake, and secretion in enterocytes [37]. It regulates the hepatic lipid metabolism by reducing lipid synthesis and induction of fatty acid β-oxidation, which probably occurs through AMPK activation. BBR played a pivotal role in decreasing liver fat content [24]. According to a meta-analysis including 2569 participants, BBR reduced TG, TC, and LDL concentrations and increased HDL levels [39]. The lipid-lowering effects of BBR seem to be more favorable than the conventional lipid-lowering medicines due to their low toxicity [38,61]. Additionally, their pairing with drugs, such as statins, could improve hyperlipidemic individuals’ therapeutic efficacy and life quality. Numerous randomized clinical trials have demonstrated its lipid-lowering and IR-alleviating actions [23]. The BBR safety profile and the beneficial outcomes from combined therapy confirm its application in individuals with hyperlipidemia and those who do not meet therapeutic goals or do not tolerate statins [8,19].

### 2.7. Cardiovascular Complications in NAFLD Preventive Role of BBR

BBR is a hypotensive and cardioprotective compound that reduces blood pressure and restores endothelial homeostasis [19]. Clinical research has reported the beneficial effects of BBR in endothelial dysfunction via regulating reactive oxygen species (ROS)/NO balance [8]. BBR promoted endothelium-dependent relaxation mediated by increasing endothelial NO release [19]. BBR application also exhibited protective effects against oxLDL-caused endothelial damage [8]. Clinical evidence suggests that BBR’s capability to reduce endothelial inflammation improves vascular health in individuals affected by CVD. The available evidence indicates a possible use of BBR in managing chronic cardiometabolic disorders [23]. Meta-analysis proved that lifestyle intervention combined with BBR lowers blood pressure more effectively than lifestyle intervention or placebo alone [39]. BBR combined with the oral antihypertensive drug also reduced blood pressure to a greater extent than the same antihypertensive drug [39]. BBR may also function as a calcium channel blocker to lower high blood pressure and increase vascular elasticity [19]. The CV effects of BBR seem to be mediated through the AMPK [19]. BBR inhibits the intracellular accumulation of ROS, cellular apoptosis, and inflammation that contribute to vascular damage, which confirms the vasoprotective actions of BBR [19].

### 2.8. BBR Affects Gut Microbiota

The regulation of GM is another relevant target of BBR. The rise of gut Akkermansia may be associated with the cardiometabolic protective effects of BBR [60]. BBR affects GM, decreases fat absorption, and reduces inflammation by reducing exogenous antigens and enhancing SCFAs in the gut. It regulates gut barrier integrity. BBR reverts damaged tight junctions in the intestinal epithelium, thereby repairing permeability. There is a need to uncover an exact immunological mechanism for better understanding. BBR modulates hepatic lipid metabolism by direct GM modulation. BBR could trigger the secretion of GM-derived active metabolites such as butyrate. Promoting butyrate production in GM appears to be an essential mechanism of BBR in the regulatory process of energy metabolism. BBR clinically lowers glucose concentration via complex mechanisms. One of the possible paths is its effect on the SCFAs of the GM [62,63]. BBR modulated serum levels of the gut hormones associated with glucose regulation and energy homeostasis, likely interfering with the GM’s composition [64]. BBR exerts its lipid-lowering effect through modulation of the gut microbiome and attenuation of hepatic steatosis [6,8].

### 2.9. Hepatic Markers Improvement

Abnormalities in laboratory tests often might be a sign of NAFLD. The most common test results are elevated alanine transaminase (ALT) and aspartate transaminase (AST). The ratio of AST/ALT is usually less than one but may increase as the severity of the liver damage increases. BBR exerts positive effects on NAFLD-associated parameters [12]. BBR has been reported to suppress NAFLD as it lowers ALT and AST levels in T2D patients [37]. Clinical findings have shown that the effects of BBR are associated with the improvement of levels of indirect markers of hepatosteatosis (Hepatic steatosis Index, Lipid Accumulation Product) [12].

## 3. Conclusions

BBR appears to be a promising therapeutic agent for NASH as it targets multiple pathways. The progression is closely linked to inflammatory condition, obesity, IR, and metabolic syndrome [6]. BBR can also inhibit oxidative stress and an inflammation in the liver, thus preventing NASH progression [6,24,40,44,46,58]. BBR exhibits various beneficial effects on cardiovascular and metabolic diseases as it can alleviate NAFLD by regulating multiple metabolic pathways and reducing inflammation response [65].

The complex and diverse pharmacological effects of BBR, such as regulation of lipid and glucose metabolism and increased insulin sensitivity, may elucidate its promising role in the treatment of NAFLD [57]. Its pleiotropic effect positively impacts many aspects of metabolic and cardiovascular comorbidities [57].

## Data Availability

Not applicable.
